# Predicting Independence 6 and 18 Months after Ischemic Stroke Considering Differences in 12 Countries: A Secondary Analysis of the IST-3 Trial

**DOI:** 10.1155/2021/5627868

**Published:** 2021-07-30

**Authors:** André Vieira, Patrícia Soares, Carla Nunes

**Affiliations:** ^1^NOVA National School of Public Health, Public Health Research Centre, Universidade NOVA de Lisboa, Portugal; ^2^Comprehensive Health Research Centre, Universidade Nova de Lisboa, Portugal

## Abstract

**Objectives:**

This study is aimed at identifying the best clinical model to predict poststroke independence at 6 and 18 months, considering sociodemographic and clinical characteristics, and then identifying differences between countries.

**Methods:**

Data was retrieved from the International Stroke Trial 3 study. Nine clinical variables (age, gender, severity, rt-PA, living alone, atrial fibrillation, history of transient ischemic attack/stroke, and abilities to lift arms and walk) were measured immediately after the stroke and considered to predict independence at 6 and 18 months poststroke. Independence was measured using the Oxford Handicap Scale. The adequacy, predictive capacity, and discriminative capacity of the models were checked. Countries were added to the final models.

**Results:**

At 6 months poststroke, 35.8% (*n* = 1088) of participants were independent, and at 18 months, this proportion decreased to 29.9% (*n* = 747). Both 6 and 18 months poststroke predictive models obtained fair discriminatory capacities. Gender, living alone, and rt-PA only reached predictive significance at 18 months. Poststroke patients from Poland and Sweden showed greater chances to achieve independence at 6 months compared to the UK. Poland also achieved greater chances at 18 months. Italy had worse chances than the UK at both follow-ups. *Discussion*. Six and eight variables predicted poststroke independence at 6 and 18 months, respectively. Some variables only reached significance at 18 months, suggesting a late influence in stroke patients' rehabilitation. Differences found between countries in achieving independence may be related to healthcare system organization or cultural characteristics, a hypothesis that must be addressed in future studies. These results can allow the development of tailored interventions to improve the outcomes.

## 1. Introduction

Stroke is a worldwide problem with an increasing number of cases [1]. Every year, about 16 million people are affected for the first time by a stroke, of which 5.7 million die [2]. The number of individuals affected by a stroke is increasing over the years, due to population ageing. It was estimated that 1.1 million strokes occurred in 2000 and 13.7 million in 2016 [3]. Recent projections estimated that by 2030, around 77 million strokes will occur worldwide [2, 4]. Brain diseases are the major contributors to the burden associated with morbidity in Europe, with stroke being the 4th most influential disease [5].

Achieving functional independence is one of the main goals for all those involved with individuals that suffered a stroke [6, 7]. The impact of the dependent person due to stroke goes far beyond their functional activities, including costs of care borne by governments and the burden for family members or caregivers. Often, these are not only responsible for direct assistance in the physical activities of daily life but also for the management of the various biopsychosocial dimensions of the person with stroke [8]. Prediction of functional independence poststroke is mainly based on instruments as Barthel Index and Functional Independence Measure, rating functional independence in specific activities of daily-living (ADL) as feeding, grooming, or toileting [9]. However, the Oxford Handicap Scale (OHS) is a generic instrument related to overall independence and overall disability in life, which tries to differentiate an independent person from a dependent person [10]. This scale is aimed at assessing the general level of handicap of the respondent in his/her lifestyle and that may state different outcomes from the functional independence measures reported.

The European Stroke Organization action plan between 2018 and 2030 considers poststroke rehabilitation an important target [11]. Rehabilitation is instrumental in enabling people with functional limitations to remain in or return to their home or community, live independently, and participate in education, work, and civic life. Yet, it is expected that functional status of independence status 3-6 months after stroke does not change considerably in a large proportion of individuals, even though transitions between (ADL) independence and dependency can occur up to 5 years after stroke [12]. So, most patients may have discharged from unit cares to community care 6 months after stroke and other factors beyond age and severity may also influence the independence transitions [13, 14].

In addition to the clinical and inherent factors of the individual, there are potential differences in content and organizational aspects of rehabilitation between national or regional systems that may facilitate or prevent the enhancement of the independence of poststroke individuals in the community after months or years [15]. For example, patients in low-income and middle-income countries have poorer access to stroke services, which may leads to lower access to other rehabilitation services and higher survival rates with severe dependency [16]. Also, a lack of clinical guidelines to aid clinical decision-making about access to stroke rehabilitation lead to subjective approaches by patient-level and organizational factors alongside clinicians' characteristics across services and countries [17]. The process of care involved in patient's reintegration from hospital to the community (rehabilitation and general practice) is frequently unsuccessful, regardless of the country. Nevertheless, since the professional hierarchy of stroke teams varies with the national context, healthcare systems may also differ on the provided treatments and in the clinical pathways for the transition to the community [16].

The development of medium/long-term models of independence prognosis can assist clinicians in adapting rehabilitation, referral, and discharge planning for poststroke users, reducing health-related costs and the associated economic impact [18–20]. Factors such as age and poststroke severity are well known as predictors of functional status and, therefore, patient's independence [19, 21]. The size of the vascular lesion, the presence of atrial fibrillation, the ability to walk, and the functional state before the stroke have also been included in some studies [21]. Predictors of functionality and mortality in individuals affected by a stroke have the attention of researchers since the 1980s, with a substantial increase in the number of related studies [22]. By 2018, more than 195 predictive variables have been identified for poststroke functional recovery, and at least more than 63 multivariate models of functionality prognosis have been found [9, 23]. However, choosing a specific prognostic model remains a controversial topic, since there is hardly a single model for all situations, subgroups, or times of assessment. It is essential, as early as possible, to predict which factors are associated with a greater chance of independence in the medium and long term, to allocate personalized resources for each subgroup. Furthermore, the odds of obtaining independence could be different among countries due to their different healthcare systems and rehabilitation methods. However, the literature is scarce regarding differences between countries and long-term predictors of independence.

Hence, this study is aimed at identifying the best clinical model to predict poststroke independence at 6 and 18 months, considering sociodemographic and clinical characteristics, and then identifying differences between countries.

## 2. Methods

### 2.1. Sample and Design of the Original Study

The information used for analysis was obtained from the database provided by the study “International Stroke Trial-3” (IST-3) [24], a resource provided to carry out secondary analyzes [25]. The original study was a randomized controlled multicenter study that collected information from 3035 subjects with ischemic stroke, in 156 centers in 12 different countries between 2000 and 2015. The primary objective of IST-3 was to verify whether the administration of activator of recombinant intravenous tissue plasminogen (rt-PA) increased the proportion of independent persons at 6 months, in the intervention group. Additionally, follow-ups were also performed at 18 months poststroke. All methods and procedures used for data collection can be accessed in the IST-3 protocol [24]. Briefly, the selection criteria for integration in the study were having symptoms and clinical signs of acute stroke, precise knowledge of when the stroke appeared, the possibility of starting treatment for thrombolysis in the first 6 hours after the onset of the stroke, and guarantee of the reliability of computed tomography (CT) or magnetic resonance (MR) to exclude intracranial hemorrhages or structural brain injuries that could mimic a stroke. CT and MRI were performed before the randomization of the participants and repeated 24 and 48 hours after the occurrence of the stroke, which can also be repeated if there was suspicion of neurological deterioration in the first 7 days. The sample size of the study was recalculated in 2007 for 3100, which gave 80% power to detect an absolute difference of 4.7% in the primary outcome [26]. The protocol was approved by the Multicenter Research Ethics Committees, Scotland (MREC/99/0/78), and by local ethics committees in 30 centers in different countries.

### 2.2. Definition of Variables

The outcome variable is “Independence” at 6 and 18 months. In the IST-3 study, the Oxford Handicap Score (OHS) scale of 6 scoring levels was used to measure the degree of independence at 7 days, 6 and 18 months after the stroke, which is an autoreported scale variant of the modified Rankin scale commonly used [10]. Scores between 0 and 2 on the OHS scale were recoded to categorize the event as “Independent” and between 3 and 5 as “dependent.” [27, 28] Scores of 6 (“Death”) were excluded from the analysis. The clinical covariables collected in the baseline (recorded up to a maximum of 6 hours immediately after the onset of stroke symptoms) were classified as age (categorized in classes of 10 years), gender (male/female), previous history of transient ischemic attack/stroke (yes/no), atrial fibrillation (yes/no), living alone before the stroke (yes/no), degree of severity immediately after the stroke through the scale *National Institutes of Health Stroke Scale* (NHISS) (classified as 1-4 mild, 5-15 mild to moderate, 16-20 mild, 21-42 severe), stroke subtype (anterior total circulation strokes (TACI), anterior partial circulation strokes (PACI), lacunar infarcts (LACI), posterior circulation infarctions (POCI), or others), intervention with rt-PA (yes/no), ability to raise both upper limbs (yes/no), and ability to walk alone (yes/no). Previous state of independence was not included because more than 99.9% were independent. Finally, a geodemographic variable was also included, relative to the country where the subject was hospitalized. Collaborating countries were Austria, Australia, Belgium, Canada, Italy, Mexico, Norway, Poland, Portugal, Sweden, Switzerland, and UK (Austria, Canada, Mexico, and Switzerland data were grouped to “Others,” due to the low number of participants).

### 2.3. Data Analysis

Descriptive analysis was performed through absolute and relative frequencies. Due to the high number of dropouts between 6 and 18 months, the answers between those who completed the questionnaires up to 18 months and 6 months were compared. All categorical covariates were tested using the chi-square test (results in Table 1) [29]. A statistical significance of *p* < 0.05 was considered for every analysis.

The odds ratio (OR) and respective confidence intervals (CI) of all clinical covariates were estimated for the subject's independence at 6 and 18 months, individually, through bivariate logistic regression (RL), for a descriptive purpose only (results on Table 2).

The model was developed in several steps. First, all covariates were adjusted for age and severity due to their clinical relevance for the outcome [9, 30]. Gender was not adjusted to the model since it was not associated with poststroke independence in a systematic review [9]. Finally, an automatic stepwise regression procedure was used, using the forward LR selection method [29], to select statistically significant variables, after adjustment for age and severity, for 6 and 18 months (considering exclusion criteria *p* > 0.10). Odds ratio with 95% CI was used as the measure of association. The adequacy and predictive capacity of the models were assessed using the Hosmer-Lemeshow adjustment test, the Nagelkerke R2 test, and the area under the curve (AUC) of receiver operation characteristic (ROC) [29].

To ascertain differences between countries, the geographical variable was added to the final predictive models. The UK was used as the class reference since it is the country with a higher number of participants. The adequacy and predictive capacity of the model were then rechecked.

## 3. Results

A total of 3035 subjects, most with ages between 71 and 90 years old (70.3%), were included in the analysis, with 51.7% (*n* = 1570) being male. The characteristics of the sample are described in Table 3.

At 6 months 35.8% (*n* = 1088) of the participants were independent, 37.3% (*n* = 1132) were dependent, and 815 died (26.9%). There were no dropouts. At 18 months, 17.6% (*n* = 533) people were lost to follow-up and 35.4% (*n* = 1075) died. The proportion of independents decreased to 29.9% (*n* = 747) and dependents to 22.4% (*n* = 680). Of those who completed follow-up at 18 months, about 5.7% (*n* = 82) were dependent at 6 months and became independent, and the reverse was observed in 8.3% (*n* = 119).

**Figure 1:**
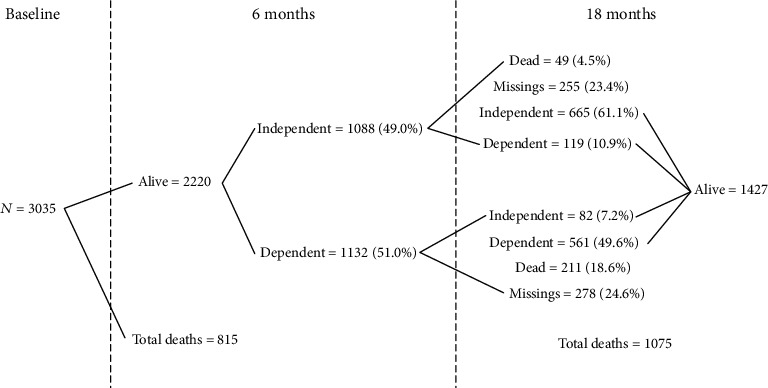
Baseline (*n* = 3035), 6 months (*n* = 3035), and 18 months (*n* = 2502).

The transitions of functional independence status from 6 to 18 months can be seen in Figure 1.

Table 1 presents the comparison between all valid answers at 18 months and the baseline sample (or 6 months, once there were no dropouts at this follow-up). We found a difference in the distribution of responses for the variable countries (*p* value ≤ 0.01) and “Able to lift both upper arms” (*p* value = 0.04) among the two samples.

Bivariate analysis for each clinical covariate related to independence can be found in Table 2.

In Table 4, we can see the multivariate analysis of each clinical covariate adjusted for age and severity at 6 and 18 months: gender, living alone, and intervention with rt-PA were not identified as statistical predictors of independence at 6 months but were statistically significant at 18 months.

### 3.1. A Predictive Model of Poststroke Independence at 6 Months and 18 Months

Table 5 shows final prediction models for independence at 6 months and 18 months.

At 6 months, six subjects (<0.1% relative to the baseline sample) were excluded from the analysis due to incomplete data (5 lacked information on the previous history of TIA/CVA, and one was excluded for being the only patient with NHISS value = 0). Gender, living alone, and intervention rt-PA were not included in the final model because they have not reached statistical significance in the analysis adjusted to age and severity (Table 4). Atrial fibrillation was the only variable ruled out in the final model.

This model was adequate, fitted to Hosmer-Lemeshow test (*p* = 0.965), and with fair discrimination (0.307 for Nagelkerke, AUC = 0.782).

At 18 months, from the initial sample, 539 subjects (17.8% relative to the baseline sample) were excluded from the analysis due to missing data in some variables. Except for the previous history of TIA/CVA, all other variables were statistically significant when adjusted to age and severity (Table 4) and therefore included in the final model. Similar to the model for 6 months, only atrial fibrillation was excluded in the final model.

This model was adequate, fitted to Hosmer-Lemeshow test (*p* = 0.789), and with fair discrimination (0.294 for Nagelkerke, AUC = 0.774).

In both final models at 6 and 18 months after stroke, age and NHISS have an OR above 6 (although the CI has a large amplitude, which makes the point estimate imprecise).

### 3.2. Relationship of Countries in Predicting Poststroke Independence

Table 6 presents the results of the addition of the variable “Countries” as a predictor for independence in the final models.

All the covariables added in the final models with countries remained statistically significant after the inclusion of countries, except for “Living Alone” at 18 months. Models at 6 and 18 months showed good values at the Hosmer-Lemeshow tests (*p* = 0.955 and *p* = 0.784, respectively), Nagelkerke (0.322 and 0.308, respectively), and fair discrimination values in AUC (0.789 and 0.781, respectively).

## 4. Discussion

The purposes of this study were to create the best predictive models with available variables to identify differences between countries for poststroke independence in alive patients. In this study, we developed predictive models for poststroke subject's independence at 6 and 18 months, based on clinical characteristics collected within 6 hours after the stroke. Age, severity, stroke subtype, ability to raise both arms, and ability to walk were predictors for poststroke independence at both follow-ups. The previous history of TIA/stroke was also for independence at 6 months after stroke. On the other hand, living alone before stroke, rt-PA intervention, and gender were also predictors of independence in the final model 18 months after stroke. Considering countries, Poland had more chances for achieving independence after the stroke compared to the UK at 6 and 18 months after stroke, Sweden also but only at 6 months (although IC95% at 18 months is suggestive to be explored in other future studies), and Italy demonstrated fewer chances of achieving independence at 6 and 18 months after stroke.

Age, severity (measured by NHISS), and subtype were predictive of poststroke independence both at 6 and 18 months. These characteristics are usually found in predictive models and are well accepted as explaining the success/failure for functional recovery [23]. Age and severity were described as the main factors in predicting functionality in the literature [9]. Other factors found in our analysis, such as the history of TIA/stroke, raising both arms of the bed, and walking alone, are also described in the literature [9, 31], but less frequently achieves statistic relevance when modelled with other variables such as age and severity [32].

In our study, gender, rt-PA intervention, and living alone before stroke were not statistically significant in the final model at 6 months, but at 18 months. In some studies [33, 34], females appeared to have worse functional outcomes after stroke; however, gender is not included in many predictive models in the literature and its relevance is not consensual [23]. Our analysis found that gender may influence independence poststroke only 18 months after the event but not at 6, which may contribute to the controversy found in literature, although at 18 months we also found worse changes for women to achieve independence. There is some controversy regarding the predictive impact of the intervention, rt-PA, which has been associated with better outcomes of survival at early stages, at longer stages [32, 35, 36]. Similarly, to our results, other studies found no statistical association for this intervention in predicting functionality at 6 months [32]. Thus, predictive relevance found in our study only at 18 months may be an important indicator when considering future research with rt-PA, not only to predict survival or short-term independence [30] but also for long-term independence. This may be an important finding since some countries still struggle with its implementation [37]. A study [31] found no relationship between poststroke independence and living alone at 3 and 12 months, although this variable is not taken into account in several prediction models analyzed in a systematic review [9]. However, studies from Craig et al. and Rejnö et al. found contradictory odds favouring better functional outcomes for individuals living alone in a range of time between 72 hours and 5 years of follow-up, being the longest follow-up negatively related to living alone [12, 38]. We found a positive association between living alone and independence achievement at 18 months, which may suggest that a set of experiences functioning alone may help the individual to regain independence more effectively in the long term.

Determining chances for recovery of independence provide levels of specificity and expectations for health professionals involved in the rehabilitation of poststroke users, after validation. It is an important contributor for anticipating the schedule of rehabilitation and/or social integration services, individually. Early prediction of long-term care levels may lead to early supported discharge, improve the efficiency of stroke management, and assist in healthcare planning [39]. To our knowledge, this study used an uncommon combination of variables to predict overall independence at 18 months, since most models use functional independence criteria over generic independence, measured by OHS. Previous studies [32, 40–42] found predictive models with discriminative capacities ranging from 0.79 to 0.90 AUC for 6 months. Thus, the model's value obtained in our study (AUC = 0.78) is lower than these values found in the literature, but none of those studies were multicentric or had more than one country participating. In an analysis carried out based on the same individuals from the original study, but with a much lower number of participants, the AUC values were higher to those found in this study for 6 months but, despite using different variables such as being independent before the stroke and the Glasgow scale, they also divided the analysis in being alive and independent vs. being alive and dependent or being dead, which was different from our study, that only considered alive patients [43]. For 18 months poststroke, no studies were found to predict independence, as longer follow-ups for this outcome (apart from functionality) do not abound in the literature [44]. The closest study found for 12 months after a stroke involving more than one country found a model with an AUC of 0.88; however, this study included several types of stroke (hemorrhagic/ischemic), not just ischemic [31].

As found in the patients in this study, bidirectional transitions of independence may occur in some cases over time. It is known that physical therapy, 6 months after stroke, may not inflict more independence than before [45]. Anyways, the type of physical therapy intervention may be important in changing these results. One study found that users who were independent at 3 months poststroke can become dependent within one year, an overall deterioration rate of this transition of 3% a year [12]. Factors such as age, NIHSS score at baseline, diabetes, and self-perceived unmet care needs were related with patients transitions from ADL independence to dependency 3 months after stroke and onwards, while the opposite (from dependent to independent 3 months after stroke) was predicted by factors as living alone before the stroke, being an ischemic stroke, and the severity rated by NIHSS score at baseline. We can hypothesize that dependent patients at 6 months undergo more therapy than independent patients, which may contribute to their transition to independence at 18 months. Or the opposite, independent patients at 6 months may leave the intervention which contributed to that. However, given the aging of the sample under study, the transition from independent states to dependents is more easily understood, and the stroke may or may not enhance this process.

Studies on differences between countries (or national health systems) for predicting independence in the poststroke subject are scarce. The European Stroke Organization is defined as a research priority the efficient implementation of long-term rehabilitation strategies and improves participation and integration into society among stroke survivors [11]. At 6 months, between 10 and 26% of ischemic stroke survivors in the UK and USA are expected to be institutionalized into long care facilities [13]. In Poland, about 96.1% of stroke survivors were at their home alone or with family at 6 months and 95.6% at 18 months [14]. About 24.6% were in rehabilitation programs at 6 months and 19.3% were at 18 months. This was similar to other studies in Sweden [46] with 92% and 88% of stroke survivors living in their homes at 6 and 12 months and another [47] involving the UK and Australia, where 75.3% of poststroke patients less than 65 years were living at home at 12 months.

In our study, it seems that patients from Sweden and Poland have greater chances to achieve independence at 6 months when compared with similar patients from the UK. Individuals living in Poland had nearly two times more chances of independence after a stroke than individuals living in the UK. This difference might also be attributed to healthcare organization and quality and not only to variations in age or severity. The Nordic countries are recognized as having one of the best healthcare systems in the world, with structural differences in stroke rehabilitation care, incorporating multidisciplinary therapies after discussions involving patients, families the medical team, and experts [48, 49]. In a study considering 28 European countries, Italy was the 4^th^ country with the highest healthcare costs per person who suffers a stroke, the UK was the 7^th^, Sweden was below the top 10, and Poland was at 20^th^ place [50]. In our study, the chances to achieve independence after stroke in each country are reverse to the economic government's health care investment, and this should be explored in future studies. The effectiveness of different rehabilitation systems should be investigated in different sociocultural contexts and explore which particularities in each set may have a role in achieving individual independence. Thereby, contributing to cost-effectiveness programs which are important for economic and public health development policies [50]. Countries with better chances of independence poststroke may have similar characteristics that should be explored and deeply understood, other than clinical variables, or direct health care cost. Long-term rehabilitation has been addressed as a vital issue in stroke needs across many countries in Europe [51], including the UK, but perhaps social reintegration and rehabilitation programs after acute care play a major role in the achievement of independence poststroke. Examples are the support offered by healthcare systems to regain social participation [52] or return to work [53]. Future studies should also investigate differences between regional and national healthcare systems and explore the factors for success.

The choice of the outcome variable requires some caution in its analysis. The OHS uses the term “handicap” instead of “disability,” from the original scale, the modified Rankin Scale. This was meant to include additional important poststroke handicaps other than mobility [10]. Despite being closely linked to the physical recovery of the poststroke subject, the scale's score also reflects other dimensions not directly related to physical or cognitive recovery, such as the lifestyle and social dimension [10]. This predictive study on independence differs from the majority of other research found, where conceiving predictive models poststroke were strictly based on functional independence scales [23].

Nevertheless, the uneven distribution of the sample among the countries can strongly contribute to the lack of significance of differences found with other countries so this study should be replicated. Recent data should be collected to ascertain whether these differences persist and explore possible reasons as to why this is happening, where qualitative data could give more insights into other contextual factors involved. Also, before clinical application, the proposed predictive models should be internally validated and replicated in other independent samples (external validation) to verify their suitability.

In the information collected through the database, one of the factors that could be relevant for analysis may be the fact that the subject was or was not independent previously before the stroke. However, this was not included due to the huge discrepancy in the answers given (*n* of yes <0.01%). Also, the fact that there are characteristics with a predictive value that was not collected in the initial sample (as economic status, comorbidities, cognitive status, or education level) may also limit the analysis, since only predominantly known factors with a possible relationship with poststroke functionality and independence were used.

These findings thus contribute to help planning other studies hypothesizing that clinical services, environments, and cultural contexts between countries may alter the chances of achieving poststroke independence. Therefore, identify the particularities of each healthcare system, how they work with the reintegration in the community, and what strategies are used for achieving poststroke patient's independence across different regions and countries should be specifically investigated in the future.

## Figures and Tables

**Table 1:** 

Characteristics of sample participants vs. sample at 18 months	Baseline sample 3035, *n* (%)	18 months 2502, *n* (%)	*p* value
Age			0.95
18-50	127 (4.2%)	93 (3.7%)		
51-60	202 (6.7%)	159 (6.4%)		
61-70	365 (12.0%)	306 (12.2%)		
71-80	724 (23.9%)	596 (19.6%)		
81-90	1407 (46.4%)	1170 (46.8%)		
> 91	210 (6.9%)	178 (7.1%)		
NHISS			0.35
0 (no clinical signs)	1 (0.1%)	1 (0.1%)	
1-4 (light)	399 (13.1%)	306 (12.2%)	
5-15 (mild to moderate)	1665 (54.9%)	1341 (53.6%)	
16-20 (moderate)	543 (17.9%)	476 (19.0%)	
21-42 (severe)	427 (14.1%)	378 (15.1%)	
Stroke subtype			0.47
TACI	1306 (43.0%)	1129 (45.1%)	
PACI	1146 (37.8%)	913 (36.5%)	
LACI	332 (10.9%)	275 (11.0%)	
POCI	246 (8.1%)	180 (7.2%)	
Other	5 (0.2%)	5 (0.2%)	
Lived alone before the stroke			0.73
Yes	1129 (37.2%)	942 (37.6%	
No	1906 (62.8%)	1560 (62.4%)	
Previous history of TIA/stroke			0.81
Yes	699 (23.0%)	583 (23.3%)	
No	2331 (76.8%)	1914 (76.5%)	
Unknown	5 (0.2%)	5 (0.2%)	
Atrial fibrillation			0.49
Yes	914 (30.1%)	755 (31.0%)	
No	2121 (69.9%)	1727 (69.0%)	
Intervention			0.93
rt-PA	1515 (49.9%)	1246 (49.8%)	
Control	1520 (50.1%)	1256 (50.2%)	
Able to raise both arms			0.04
Yes	1351 (44.5%)	1044 (41.7%)	
No	1684 (55.5%)	1458 (58.3%)	
Able to walk without help			0.14
Yes	487 (16.0%)	365 (14.6%)	
No	2548 (84.0%)	2137 (85.4%)	
Country			<0.01
Australia	179 (5.9%)	177 (7.1%)	
Belgium	73 (2.4%)	70 (2.8%)	
Italy	326 (10.7%)	236 (9.4%)	
Norway	204 (6.7%)	199 (8.0%)	
Others^∗^	80 (8.4%)	58 (2.3%)	
Poland	347 (11.4%)	306 (12.2%)	
Portugal	82 (2.7%)	38 (1.5%)	
Sweden	297 (9.8%)	296 (11.8%)	
United Kingdom	1447 (47.7%)	1122 (44.8%)	

NHISS: National Institutes of Health Stroke Scale; TACI: strokes of the anterior total circulation; PACI: infarctions of anterior partial circulation; LACI: lacunar infarcts; POCI: posterior circulation strokes; TIA/AVC: transient ischemic attack/stroke; rt-PA: recombinant intravenous tissue plasminogen activator. ^∗^Others—including Austria, Canada, Mexico, and Switzerland.

**Table 2:** Predictors for independence at 6 and 18 months poststroke, with OR and 95% CI.

	Independence at 6 months		Independence at 18 months	
	Crude OR (95% CI)	*p*	Crude OR (95% CI)	*p*
Age (reference class: >91)		<0.01		<0.01
18-50	7.35 (4.06–14.00)	<0.01	5.49 (2.51-12.02)	<0.01
51-60	5.71 (3.34-9.77)	<0.01	5.10 (2.47-10.53)	<0.01
61-70	4.22 (2.56-6.94)	<0.01	3.52 (1.78-6.97)	<0.01
71-80	2.76 (1.72-4.44)	<0.01	2.07 (1.07-4.01)	0.03
81-90	2.11 (1.33-3.35)	<0.01	1.83 (0.95 – 3.51)	0.07
Gender (reference class: female)				
Male	1.55 (1.31-1.83)	<0.01	1.72 (1.40-2.13)	<0.01
NHISS (reference class: severe (21–42))		<0.01		<0.01
Mild (1–4)	34.46 (20.46-58.03)	<0.01	21.93 (11.51-41.80)	<0.01
Moderate (5–14)	7.02 (4.55-11.40)	<0.01	6.32 (3.52-11.35)	<0.01
Moderate to severe (15–20)	2.00 (1.18-3.35)	0.09	1.50 (0.77-2.92)	0.23
Previous TIA/stroke history (reference class: yes)				
No	1.28 (1.05-1.57)	0.02	1.41 (1.14-1.75)	<0.01
Stroke subtype (reference class: TACI)		<0.01		<0.01
PACI	3.62 (2.94-4.45)	<0.01	3.09 (2.4-4.00)	<0.01
LACI	5.41 (4.06-7.20)	<0.01	5.64 (3.96-8.02)	<0.01
POCI	6.12 (4.38-8.55)	<0.01	5.16 (3.38-7.87)	<0.01
Other	4.12 (0.68-24.81)	0.12	3.40 (0.562-20.59)	0.18
Lived alone before the stroke (reference class: no)				
Yes	1.02 (0.86-1.22)	0.80	0.94 (0.76-1.17)	0.60
Intervention with rt-PA (reference class: no)				
Yes	1.09 (0.92-1.28)	0.33	1.25 (1.01-1.54)	0.04
Atrial fibrillation (reference class: yes)				
No	1.80 (1.48-2.19)	<0.01	1.92 (1.49-2.47)	<0.01
Able to lift both upper limbs (reference class: no)				
Yes	4.52 (3.78-5.41)	<0.01	4.15 (3.32-5.17)	<0.01
Able to walk without help (reference class: no)				
Yes	5.21 (4.08-6.65)	<0.01	4.06 (3.04-5.43)	<0.01
Country (Reference class: UK)				
Australia	1.14 (0.79-1.65)	0.48	0.97 (0.65-1.46)	0.88
Belgium	1.42 (0.81-2.48)	0.22	1.74 (0.92-3.28)	0.08
Italy	0.99 (0.75-1.31)	0.95	0.78 (0.54-1.11)	0.17
Norway	1.38 (0.99-1.94)	0.06	1.49 (1.02-2.17)	0.04
Others^∗^	2.18 (1.26-3.81)	<0.01	1.14 (0.55-2.37)	0.73
Poland	3.05 (2.28-4.08)	<0.01	2.92 (2.04-4.16)	<0.01
Portugal	0.71 (0.41-1.24)	0.23	0.57 (0.14-2.30)	0.43
Sweden	1.66 (1.25-2.20)	<0.01	1.51 (1.10-2.08)	0.01

NHISS: National Institutes of Health Stroke Scale; TACI: strokes of the anterior total circulation; PACI: infarctions of anterior partial circulation; LACI: lacunar infarcts; POCI: posterior circulation strokes; TIA/AVC: transient ischemic attack/stroke; rt-PA: recombinant intravenous tissue plasminogen activator. ^∗^Others—including Austria, Canada, Mexico, and Switzerland.

**Table 3:** Characteristics of the survival participants.

	Baseline Sample (*N* = 3035), *n* (%)	6 months (*n* = 2220), *n* (%)		18 months (*n* = 1427), *n* (%)	
		Independence	Dependence	Independence	Dependence
Age					
18-50	127 (4.2%)	88 (69.3%)	35 (27.6%)	62 (66.7%)	25 (26.9%)
51-60	202 (6.7%)	125 (61.9%)	64 (31.7%)	99 (62.3%)	43 (27.0%)
61-70	365 (12.0%)	186 (51.0%)	129 (35.3%)	148 (48.4%)	93 (30.4%)
71-80	724 (23.9%)	278 (38.4%)	294 (40.6%)	185 (31.0%)	198 (33.2%)
81-90	1407 (46.4%)	385 (27.4%)	534 (38.0%)	239 (20.4%)	290 (24.8%)
> 91	210 (6.9%)	26 (12.4%)	76 (36.2%)	14 (7.9%)	31 (17.4%)
NHISS					
0 (no clinical signs)	1 (0.1%)	—	—	—	—
1-4 (mild)	399 (13.1%)	313 (78.4%)	64 (16.0%)	217 (70.9%)	53 (17.3%)
5-15 (mild to moderate)	1665 (54.9%)	684 (41.1%)	669 (40.2%)	474 (35.3%)	402 (30.0%)
16-20 (moderate)	543 (17.9%)	69 (12.7%)	244 (44.9%)	42 (8.8%)	150 (31.5%)
21-42 (severe)	427 (14.1%)	22 (5.2%)	155 (36.3%)	14 (3.7%)	75 (19.8%)
Gender					
Female	1570 (51.7%)	474 (30.2%)	616 (39.2%)	312 (24.0%)	376 (28.9%)
Male	1465 (48.3%)	614 (41.9%)	516 (35.2%)	435 (36.2%)	304 (25.3%)
Stroke subtype					
TACI	1306 (43.0%)	203 (15.5%)	557 (42.6%)	138 (12.2%)	313 (27.7%)
PACI	1146 (37.8%)	534 (46.6%)	405 (35.3%)	356 (39.0%)	261 (28.6%)
LACI	332 (10.9%)	203 (61.1%)	103 (31.0%)	159 (57.8%)	64 (23.3%)
POCI	246 (8.1%)	145 (58.9%)	65 (26.4%)	91 (50.6%)	40 (22.2%)
Other	5 (0.2%)	3 (60%)	2 (40.0%)	3 (60%)	2 (40.0%)
Lived alone before the stroke					
Yes	1129 (37.2%)	396 (35.1%)	418 (37.0%)	278 (29.5%)	244 (25.9%)
No	1906 (62.8%)	692 (36.3%)	714 (37.5%)	469 (30.1%)	436 (27.9%)
Previous history of TIA/stroke					
Yes	699 (23.0%)	214 (30.6%)	271 (38.8%)	143 (24.5%)	159 (27.3%)
No	2331 (76.8%)	872 (37.4%)	860 (36.9%)	602 (31.5%)	521 (27.2%)
Unknown	5 (0.2%)	—	—	—	—
Atrial fibrillation					
Yes	914 (30.1%)	212 (23.2%)	343 (37.5%)	130 (16.8%)	196 (25.3%)
No	2121 (69.9%)	876 (41.3%)	789 (37.2%)	617 (35.7%)	484 (28.0%)
Intervention					
rt-PA	1515 (49.9%)	554 (36.6%)	553 (36.5%)	393 (31.5%)	320 (25.7%)
Control	1520 (50.1%)	534 (35.1%)	579 (38.1%)	354 (28.2%)	360 (28.7%)
Able to raise both arms					
Yes	1351 (44.5%)	778 (57.6%)	404 (29.9%)	524 (50.2%)	246 (23.6%)
No	1684 (55.5%)	310 (18.4%)	728 (43.2%)	223 (15.3%)	434 (29.8%)
Able to walk without help					
Yes	487 (16.0%)	357 (73.3%)	97 (19.9%)	240 (65.8%)	71 (19.5%)
No	2548 (84.0%)	731 (28.7%)	1035 (40.6%)	507 (23.7%)	609 (28.5%)
Country					
Australia	179 (5.9%)	60 (33.5%)	69 (38.5%)	52 (29.4%)	61 (34.5%)
Belgium	73 (2.4%)	27 (37.0%)	25 (34.2%)	26 (37.1%)	17 (24.3%)
Italy	326 (10.7%)	111 (34.0%)	147 (45.1%)	64 (27.1%)	94 (39.8%)
Norway	204 (6.7%)	80 (39.2%)	76 (37.3%)	77 (38.7%)	59 (29.6%)
Others∗	80 (2.6%)	35 (43.8%)	21 (26.3%)	15 (25.9%)	15 (25.9%)
Poland	347 (11.4%)	186 (53.6%)	80 (23.1%)	141 (46.1%)	55 (18.0%)
Portugal	82 (2.7%)	20 (24.4%)	37 (45.1%)	3 (7.9%)	6 (15.8%)
Sweden	297 (9.8%)	134 (45.1%)	106 (35.7%)	122 (41.2%)	92 (31.1%)
United Kingdom	1447 (47.7%)	435 (30.1%)	572 (39.5%)	247 (22.0%)	281 (25.0%)

NHISS: National Institutes of Health Stroke Scale; TACI: strokes of the anterior total circulation; PACI: infarctions of anterior partial circulation; LACI: lacunar infarcts; POCI: posterior circulation strokes; TIA/AVC: transient ischemic attack/stroke; rt-PA: recombinant intravenous tissue plasminogen activator. ^∗^Others: Austria, Canada, Mexico, and Switzerland were grouped into one group due to their small number of participants.

**Table 4:** Predictors for independence at 6 and 18 months poststroke adjusted for age and severity, with OR and 95% CI.

	Independence at 6 months		Independence at 18 months	
	Adjusted OR^∗^ (95% CI)	*p*	Adjusted OR^∗^ (95% CI)	*p*
Age (reference class: >91)		<0.01		<0.01
18-50	6.48 (3.43-12.26)^a)^	<0.01	6.17 (2.65-14.38)^a)^	<0.01
51-60	4.18 (2.35-7.42)^a)^	<0.01	4.94 (2.28-19.71)^a)^	<0.01
61-70	3.19 (1.87-5.44)^a)^	<0.01	3.47 (1.67-7.19)^a)^	<0.01
71-80	2.45 (1.48-4.06)^a)^	<0.01	2.26 (1.11-4.58)^a)^	0.02
81-90	1.86 (1.14-3.05)^a)^	0.01	1.94 (0.97-3.89)^a)^	0.06
Gender (reference class: female)				
Male	1.14 (0.95-1.38)	0.17	1.4 (1.07-1.72)	0.01
NHISS (reference class: severe (21–42))		<0.01		<0.01
Mild (1–4)	30.81 (18.20-52.15)^b)^	<0.01	20.24 (10.53-38.91)^b)^	<0.01
Moderate (5–14)	6.91 (4.35-10.99)^b)^	<0.01	6.18 (3.41-11.20)^b)^	<0.01
Moderate to severe (15–20)	1.90 (1.12-3.12)^b)^	0.02	1.40 (0.71 - 2.75)^b)^	0.33
Previous TIA/stroke history (reference class: yes)				
No	1.22 (0.97-1.53)	0.08	1.17 (0.88-1.55)	0.27
Stroke subtype (reference class: TACI)		<0.01		<0.01
PACI	1.75 (1.38-2.23)	<0.01	1.58 (1.19-2.13)	<0.01
LACI	1.98 (1.43-2.75)	<0.01	2.28 (1.53-3.39)	<0.01
POCI	2.23 (1.53-3.25)	<0.01	2.00 (1.25-3.20)	<0.01
Other	0.52 (0.08 - 3.31)	0.49	0.56 (0.09-3.62)	0.54
Lived alone before the stroke (reference class: no)				
Yes	1.12 (0.92-1.36)	0.27	1.26 (0.99-1.61)	0.06
Intervention with rt-PA (reference class: no)				
Yes	1.14 (0.95-1.38)	0.16	1.38 (1.10-1.74)	<0.01
Atrial fibrillation (reference class: yes)				
Not	1.23 (0.99-1.54)	0.07	1.33 (1.00-1.76)	0.05
Able to lift both upper limbs (reference class: no)				
Yes	2.45 (1.97-3.04)	<0.01	2.34 (1.79-3.06)	<0.01
Able to walk without help (reference class: no)				
Yes	2.52 (1.93-3.30)	<0.01	2.04 (1.48-2.83)	<0.01
Country (reference class: UK)				
Australia	1.13 (0.79-1.70)	0.55	0.93 (0.59-1.45)	0.74
Belgium	1.01 (0.54-1.90)	0.97	1.11 (0.56-2.21)	0.77
Italy	0.77 (0.57-1.04)	0.09	0.66 (0.45-0.99)	0.04
Norway	1.35 (0.93-1.95)	0.12	1.51 (1.00-2.29)	0.05
Others	2.04 (1.11-3.76)	0.02	1.06 (0.47-2.39)	0.89
Poland	2.11 (1.53-2.92)	<0.01	2.01 (1.36-2.98)	<0.01
Portugal	0.71 (0.50-1.72)	0.82	1.04 (0.23-4.78)	0.96
Sweden	1.57 (1.14-2.17)	<0.01	1.54 (1.07-2.22)	0.02

NHISS: National Institutes of Health Stroke Scale; TACI: strokes of the anterior total circulation; PACI: infarctions of anterior partial circulation; LACI: lacunar infarcts; POCI: posterior circulation strokes; TIA/AVC: transient ischemic attack/stroke; rt-PA: recombinant intravenous tissue plasminogen activator; OR: odds ratio; CI: confidence interval. ^∗^Adjusted to age and NHISS. ^a)^Odds ratio adjusted to NHISS. ^b)^Odds ratio adjusted to age.

**Table 5:** Final model for poststroke independence at 6 and 18 months.

	6 months (*n* = 2217)		18 months (*n* = 1425)	
	OR (95% CI)	*p*	OR (95% CI)	*p*
Age (reference: >91)		<0.01		<0.01
18-50	5.65 (2.93-10.87)	<0.01	6.42 (2.66-15.50)	<0.01
51-60	3.99 (2.19-7.15)	<0.01	4.90 (2.17-11.04)	<0.01
61-70	3.01 (1.73-5.19)	<0.01	3.50 (1.63-7.51)	<0.01
71-80	2.34 (1.39-3.91)	<0.01	2.35 (1.13-4.90)	0.02
81-90	1.71 (1.03-2.84)	0.04	1.89 (0.92-3.87)	0.08
Gender (reference: female)				
Male	—	—	1.44 (1.13-1.85)	<0.01
NHISS (reference: severe (21–42))		<0.01		<0.01
Mild (1–4)	7.33 (4.02-13.53)	<0.01	5.27 (2.49-11.15)	<0.01
Mild to moderate (5–15)	3.16 (1.92-5.20)	<0.01	2.99 (1.59-5.64)	<0.01
Moderate (16–20)	1.80 (1.06-3.05)	0.03	1.38 (0.70-2.73)	0.36
Stroke type (reference: TACI)		<0.01		<0.01
PACI	1.44 (1.12-1.85)	<0.01	1.30 (0.95-1.77)	0.10
LACI	2.03 (1.45-2.84)	<0.01	2.28 (1.52-3.43)	<0.01
POCI	1.76 (1.19-2.59)	<0.01	1.61 (0.99-2.62)	0.06
Others	0.41 (0.06-2.96)	0.38	0.43 (0.06-3.04)	0.40
Previous history of TIA/stroke (reference: yes)				
No	1.28 (1.02-1.61)	0.04	—	—
Intervention (reference: no)				
Yes	—	—	1.41 (1.11-1.79)	<0.01
I lived alone before the stroke (reference: no)				
Yes	—	—	1.33 (1.03-1.73)	0.03
Able to raise both arms (reference: no)				
Yes	2.10 (1.67-2.63)	<0.01	2.19 (1.65-2.91)	<0.01
Able to walk without help (reference: no)				
Yes	2.05 (1.54-2.72)	<0.01	1.69 (1.19-2.39)	<0.01

NHISS: National Institutes of Health Stroke Scale; TACI: strokes of the anterior total circulation; PACI: infarctions of anterior partial circulation; LACI: lacunar infarcts; POCI: posterior circulation strokes; TIA/AVC: transient ischemic attack/stroke; OR: odds ratio; CI: confidence interval.

**Table 6:** Differences between countries in the final models.

	6 months after stroke OR adjusted (95% CI)^∗^	*p*	18 months after stroke OR adjusted (95% CI)^∗^	*p*
*Country*		<0.01		<0.01
UK (reference)				
Australia	1.06 (0.70-1.62)	0.78	0.82 (0.51-1.31)	0.40
Belgium	0.82 (0.43-1.58)	0.55	0.98 (0.48-2.02)	0.96
Italy	0.71 (0.52-0.98)	0.04	0.64 (0.42-0.96)	0.03
Norway	1.33 (0.90-1.95)	0.15	1.46 (0.95-2.25)	0.09
Poland	1.96 (1.40-2.74)	<0.01	1.82 (1.21-2.74)	<0.01
Portugal	0.84 (0.44-1.61)	0.61	0.96 (0.20-4.64)	0.96
Sweden	1.44 (1.03-2.01)	0.03	1.39 (0.95-2.03)	0.09
Others^∗∗^	1.70 (0.90-3.20)	0.10	0.77 (0.33-1.80)	0.56

OR: odds ratio; CI: confidence interval. ^∗^Used all the variables that were obtained in the respective final models. ^∗∗^Others—including Austria, Canada, Mexico, and Switzerland.

## Data Availability

The data for this study was provided by the IST3 collaborative group and is available at University of Edinburgh DataShare: https://datashare.ed.ac.uk/handle/10283/1931.

## Additional Points

*Highlights*. (i) This study developed two models with fair predictive power for poststroke independence at 6 and 18 months based on simple and easy to collect clinical variables. (ii) We found age, gender, severity, stroke subtype, intervention with Rt-PA, living alone before stroke, and ability to raise arm and to walk as important predictors for independence for poststroke subjects at 18 months, which predictive models are sparse in the literature. (ii) Poland and Sweden demonstrate better chances for achieving independence in the predictive models at 6 compared to the UK. Poland also have at 18 months. Italy demonstrated worse outcomes at 6 and 18 months. Research is needed to understand characteristics of healthcare systems that may potentiate this outcome
